# Supplementary material for: MetaRibo-Seq measures translation in microbiomes
Source: Nat Commun. 2020 Jun 29;11:3268. doi: 10.1038/s41467-020-17081-z (PMC7324362; doi:10.1038/s41467-020-17081-z)
Supplement: Supplementary file 10 — Supplementary Data 7 [file 41467_2020_17081_MOESM10_ESM.zip › File2/Confidence_High_Taxonomy/83935_out.krona.html]

Javascript must be enabled to view this page.

members
magnitude
magnitudeUnassigned
count
unassigned
taxon
rank

83935\_out

27

27
superkingdom
2

27
phylum
1239

class
27
186801

order
27
186802

4
186803
27
family

SRS014948\_contig\_number\_16319SRS015578\_contig\_number\_contig-100\_949.159415SRS017521\_contig\_number\_28436SRS149244\_contig\_number\_contig-100\_12818.52116

841
1
genus


SRS143780\_contig\_number\_24232
1262941
1
species

572511
22
genus


SRS012902\_contig\_number\_contig-100\_485.485SRS016267\_contig\_number\_10106SRS023346\_contig\_number\_contig-100\_2655.34076SRS023829\_contig\_number\_contig-100\_6361.6361SRS042284\_contig\_number\_contig-100\_2235.44146SRS054590\_contig\_number\_contig-100\_3662.3662SRS057717\_contig\_number\_7469SRS063518\_contig\_number\_contig-100\_177.217908SRS075398\_contig\_number\_494SRS076876\_contig\_number\_contig-100\_3194.3195SRS077502\_contig\_number\_contig-100\_494.46723SRS097889\_contig\_number\_35066SRS1041095\_contig\_number\_3337SRS1041147\_contig\_number\_contig-100\_14585.14586SRS104485\_contig\_number\_contig-100\_2299.2299SRS1055067\_contig\_number\_5458SRS140645\_contig\_number\_256SRS148159\_contig\_number\_contig-100\_1754.105197SRS148511\_contig\_number\_20306SRS893253\_contig\_number\_269SRS893256\_contig\_number\_93SRS893259\_contig\_number\_5167
33038
22
species
